# Nanoscale Probing of Thermal, Stress, and Optical Fields under Near-Field Laser Heating

**DOI:** 10.1371/journal.pone.0058030

**Published:** 2013-03-28

**Authors:** Xiaoduan Tang, Shen Xu, Xinwei Wang

**Affiliations:** 1 Department of Mechanical Engineering, Iowa State University, Ames, Iowa, United States of America; 2 School of Environmental and Municipal Engineering, Qingdao Technological University, Qingdao, Shandong, P.R. China; UC Davis School of Medicine, United States of America

## Abstract

Micro/nanoparticle induced near-field laser ultra-focusing and heating has been widely used in laser-assisted nanopatterning and nanolithography to pattern nanoscale features on a large-area substrate. Knowledge of the temperature and stress in the nanoscale near-field heating region is critical for process control and optimization. At present, probing of the nanoscale temperature, stress, and optical fields remains a great challenge since the heating area is very small (∼100 nm or less) and not immediately accessible for sensing. In this work, we report the first experimental study on nanoscale mapping of particle-induced thermal, stress, and optical fields by using a single laser for both near-field excitation and Raman probing. The mapping results based on Raman intensity variation, wavenumber shift, and linewidth broadening all give consistent conjugated thermal, stress, and near-field focusing effects at a 20 nm resolution (<*λ*/26, *λ* = 32 nm). Nanoscale mapping of near-field effects of particles from 1210 down to 160 nm demonstrates the strong capacity of such a technique. By developing a new strategy for physical analysis, we have de-conjugated the effects of temperature, stress, and near-field focusing from the Raman mapping. The temperature rise and stress in the nanoscale heating region is evaluated at different energy levels. High-fidelity electromagnetic and temperature field simulation is conducted to accurately interpret the experimental results.

## Introduction

The conventional optical microscope is diffraction-limited in imaging resolution to about half of the illuminating wavelengths. Some near-field techniques can help to improve the resolution, which is achieved by placing the detector very close to the object. Scanning tunneling microscopy (STM) and atomic force microscopy (AFM), utilizing a tunneling current and force between the tip and the sample respectively, can image surfaces at the atomic level. With light probe, near-field scanning optical microscopy (NSOM) is capable of breaking the optical resolution limit by extensively employing the properties of evanescent waves [1], [2]. However, NSOM has slow scanning speed and low imaging depth and the probe must be very close to the object. Taubner *et al*. improved subwavelength imaging of buried objects by using a silicon carbide superlens based near-field microscopy [3]. The superlens used a silver slab with periodic corrugations to enhance the evanescent waves of an object and convert them into propagating waves, so that the object could be imaged in the far-field [4]. The superlens was physically placed in the near-field of an object, however. An imaging technique called stochastic optical reconstruction microscopy (STORM) provided a high imaging resolution of approximately 20 nm by controlling the fluorescence emission from a single molecule, limited only by the number of photons emitted per switch cycle [5]. The above techniques are nearsighted, as the distance between the probe and object is in the near-field. Far-field superlenses (FSLs) capable of imaging beyond the diffraction limit in the far field have been developed [6]–[9]. Transparent microspheres or photonic metamaterials were used as FSLs to reach a resolution between *λ*/3 and *λ*/14. Nanoscale solid immersion lenses (nSILs) were developed for high-quality imaging which can resolve line objects at a resolution of *λ*/2.2 [10]. However, it is hard to resolve a surface variation of objects below 50 nm with visible light. The technique employed in this work is capable of imaging a surface variation beyond the light diffraction limit in the far-field. The surface variation of an object in the horizontal plane is able to be distinguished at a resolution of *λ*/26.

Regarding nanoscale surface thermal and stress imaging, scanning thermal microscopy (SThM) has been reported as an established technique to measure nanoscale temperature distributions by attaching a temperature sensor on the apex of a tiny tip. The imaging of phonon temperature distribution for electrically heated carbon nanotube circuits was reported with a spatial resolution of ∼50 nm [11]. A near-field SThM (NSThM) operating in ultrahigh vacuum was developed to provide thermal imaging for a sample with a resolution of a few nanometers [12]. In thermometry, apertureless NSOM has been proposed by using an AFM tip or a nanoparticle. Temperature probing of silicon under AFM tip focused laser heating at a sub-10 nm scale was conducted via apertureless NSOM based on Raman thermometry [13]. Nanoscale thermal probing of graphene on 4H-SiC in the thickness direction was reported using Raman spectrometer with a resolution down to 1 nm [14]. Temperature and stress surface mapping in a doped polysilicon microheater and a silicon microcantilever has been reported at microscale using Raman spectroscopy [15]. Recently, Reserbat-Plantey *et* *al.* developed a non-invasive optical probe to provide stress mapping at nanoscale within a nanoelectromechanical system by combining Raman spectroscopy with Fizeau interferometry [16]. As Raman spectroscopy is a unique far-field technique sensitive to both temperature and stress fields [15], [17], in this paper, we use it to achieve thermal and stress surface mapping at nanoscale.

Monolayer of micro/nanoparticles is attractive due to the generation of surface textures [18]. Laser-assisted nanopatterning and nanoimprinting lithography has been proved to be able to pattern nanoscale features on a large-area substrate [19]–[21]. Pit arrays have been created on metallic surfaces under particle-enhanced laser irradiation [22]. Due to the wide application of particles, theoretical studies about optical field enhancement by micro/nano particles have been reported. Analytical calculation of a dielectric sphere under laser illumination has been performed by using the Mie scattering theory [23]. Optical field distributions of a particle on a substrate under normally irradiated laser were obtained [20], [22], [24]. Moreover, simulation of laser interaction with materials (SLIM) was used to theoretically calculate the temperature of a substrate beneath particles with laser irradiation [22], [25]. Nanoscale experimental imaging using microparticles is rarely reported. McLeod and Arnold employed a microsphere as an objective lens for nanopatterning by focusing a laser beam on a substrate [20]. Arbitrary patterns and individual features were generated with a minimum size of 100 nm and a positioning accuracy less than 40 nm. Wang *et al.* employed ordinary transparent microspheres to collect near-field object information and formed virtual images at a 50 nm resolution [6]. Light intensity distributions for solid immersion lens, a sphere and a particle on substrates were calculated and compared. In laser-assisted surface patterning using micro/nanoparticles, very high temperature rise and local stress will be induced and needed to induce surface structure change. Knowledge of the temperature and stress information in the near-field heating region under micro/nanoparticles is critical for process control and optimization. In our previous work, the temperature field inside silicon under particle induced laser heating has been measured at a subwavelength resolution [26]. To our knowledge, however, no experiment about structure imaging with temperature and stress information inside a substrate-particle system using a far-field method has been reported. Such measurement is very challenging since the near-field heating area in the substrate is very small, usually around 100 nm or smaller. Also this area is immediately below the particle, so retrieving the thermal and stress information of this region is very difficult.

In this work, far-field nanoscale mapping of conjugated thermal, stress, and near-field focusing effects in a silicon substrate beneath silica particles under laser irradiation is conducted for the first time using Raman spectroscopy at a 20 nm lateral resolution (< *λ*/26). Methodologies are developed to separate the optical, thermal, and stress effects and evaluate the temperature rise and local stress in particle-induced near-field focusing. The electromagnetic and temperature fields inside the substrate-particle system are simulated to interpret the measurement results. Our experimental work on nanoscale imaging has great potential in molecular imaging, nanolithography, nanotexturing and biomedical sensing.

## Experimental Details

### Sample Preparation

**Figure 1:**
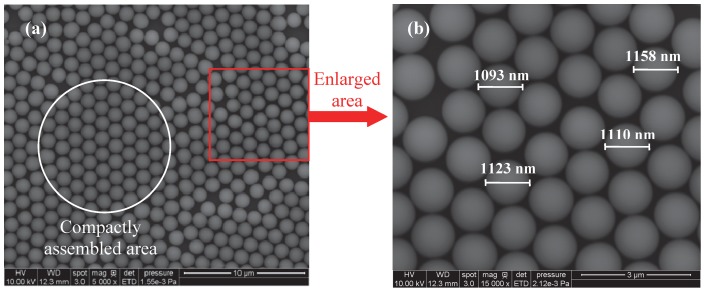
SEM images of 2-D monolayer array of silica particles assembled on a silicon wafer. The average diameter of the particles is about 1120 nm.

Silica particles are patterned on silicon wafers in a monolayer using a tilting technique [27]. Surfactant (triton-X: methanol  = 1∶400 by volume) is mixed with monodisperse silica particle suspensions [28]. The suspensions have silica particles with a solid percentage of 10% suspended in water. As-received silica spheres of 200 nm (Corpuscular), 400 nm (Polysciences), 800 nm, and 1210 nm (Bangs Laboratories) diameters are used without any surface treatment. Silicon (100) wafers (University Wafer) are cleaned in acetone and then deionized water for an hour with ultrasonic agitation. These wafers are placed on glass slides, which are tilted on a table with an angle of about 10° [27]. The mixture is dispensed onto substrates using a syringe and left to dry for about a half hour in the air. Then a 2-D monolayer of particles is formed on the substrate. Large areas of monolayer particles can be identified under a scanning electron microscope (SEM, FEI Quanta 250). Figure 1 shows typical SEM images of a silica monolayer of 1210 nm diameter assembled on silicon wafers. The compact assembled area can extend over a large area, up to 1 mm^2^, which is much larger than the laser spot area used in our experiments (∼0.5 μm^2^). The average diameter of the particles shown in Fig. 1(b) is about 1120 nm, a little smaller than the nominal diameter reported by the company.

### Nanoscale Mapping

**Figure 2:**
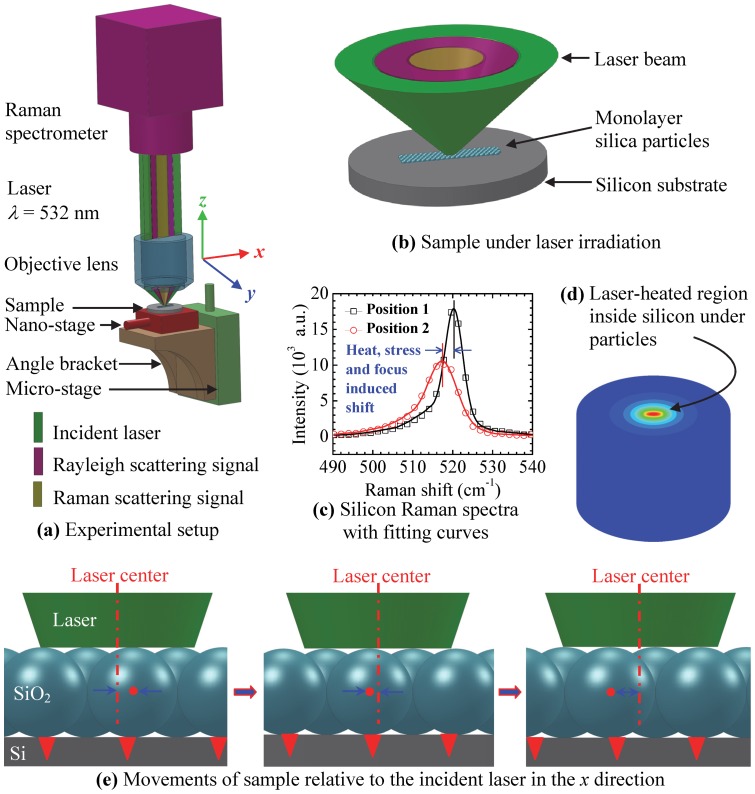
Schematic of experimental setup for far-field nanoscale imaging (not to scale). (a) A sample is located under an objective-focused laser beam from a Raman spectrometer. The movement of sample in the *x* direction is controlled by a piezo-actuated nano-stage. The focal level of the laser on the sample in the *z* direction is controlled by a motorized micro-stage. (b) The sample consists of a silicon substrate and a monolayer of silica particles. The spot size of the incident laser is about 0.5 μm in the *x*-*y* plane on a silicon substrate. (c) The Raman spectrum shifts to left due to the near-field laser heating, stress, and the out-of-focus effect. (d) The silicon substrate is heated in a sub-wavelength region (*r* ∼200 nm) right beneath the particles. (e) During the experiment, the position of the laser beam is fixed, and the sample moves along the *x* direction controlled by the nano-stage electrically without any touch of the sample and other equipment. The step of movement is 27 nm in a range of about 4 μm.

Figure 2 shows schematic of the experimental setup for the nanoscale structural imaging. The Raman scattering system consists of a confocal Raman spectrometer (Voyage^TM^, B&W Tek) and a microscope (Olympus BX51). Raman spectra are taken at room temperature by using a 532 nm laser line at variable power from 1.2–4.7 mW. The laser beam is focused by a 100× objective lens (NA  = 0.80). The movements of the sample are controlled by a piezo-actuated nano-stage (ThorLabs NFL5DP20) in the *x* direction (imaging direction) and a motorized translation stage (ThorLabs MT1-Z8) in the *z* direction. These two stages are vertically assembled together. The piezo-actuated range of the nano-stage is 20 μm with a resolution of 20 nm. The sample position in the *z* direction is adjusted by the motorized stage to change the focal level of the incident laser within a range of 12 mm and a location accuracy of 0.1 μm. The incident laser used as both Raman probing and heating source is focused on the silica particles by the objective lens. The laser is found uniformly distributed in space, and the spot size of the laser is about 500 nm, determined by using a blade method. Due to the effect of the particles, the laser beam is further focused on the silicon substrate under the particles and heats up the substrate. The size of the near-field focused area is about 200 nm. The excited Raman scattering signals and Rayleigh scattering signals are collected by using the same objective. Raman spectra of silicon substrate with silica particles on the top at different positions in the *x* direction are obtained and fitted using the Gaussian function.

The near-field heating and thermal stress inside the silicon substrate is affected by factors including the particle diameter, energy flux and focal level of the laser. The particle diameter and laser energy flux can be determined precisely before the experiments. In the experiments, it is critical to pay attention to the laser focal level in order to obtain high accuracy and repeatability of Raman signals. The position of the sample is adjusted near the focal plane within a distance of about 6 μm. Raman spectra are obtained at each level and the background noise is subtracted to obtain sound Raman signals. Raman intensity, wavenumber, and linewidth (full width at half maximum) are affected by the focal level when other environmental factors are fixed. Raman intensity and wavenumber decrease, and linewidth broadens when the sample moves away from the focal plane in the *z* direction [26].

A group of Raman spectra are obtained in the *z* direction before imaging in order to determine the focal level. The focal level is selected with the highest Raman intensity in the group. The sample is then fixed to the focal level without any vertical shift. Environmental factors such as the change of room temperature and movement of the objective would lead to a Raman spectrum difference. Therefore, the imaging process is followed immediately to minimize the effect of environmental factors. The sample is scanned along the *x* direction in a maximum range of 4.0 μm with a step of 27 or 53 nm. The movement is controlled electrically without any touch of the sample, stage, Raman spectrometer, microscope and other related equipment that would affect the quality of Raman signals. The Raman spectra change with the nanoscale movement of the sample is finally obtained. The highest energy flux is first used in the experiment, following by 79%, 50%, and 25% of the maximum energy.

### De-Conjugation of Thermal, Stress, and Optical Fields

In order to investigate the temperature rise and thermal stress caused by particle near-field focusing, experiments on silicon with particles on the surface and bare silicon are both conducted. The incident laser is first focused on the particles of the sample. The Raman spectra for silicon under monolayer silica particles are taken under four energy fluxes. Bare silicon is located around the particles in the margin of the sample. Without any movement of Raman spectrometer, the margin of the sample is moved to the laser center to obtain the Raman spectra for bare silicon. The laser is then adjusted to focus on the silicon and four energy fluxes are used as well. The integration time and measurement average are the same for those for silicon with particles. A group data of Raman spectra at different focal levels around the focal plane are obtained for each case. The Raman spectrum with the highest intensity is selected to represent each result. By using this method, the differences between the environmental situations for both bare silicon and silicon with particles are suppressed to the minimal level.

## Results and Discussion

### Nanoscale Mapping for Near-Field Heating under 1210 nm Particles

**Figure 3:**
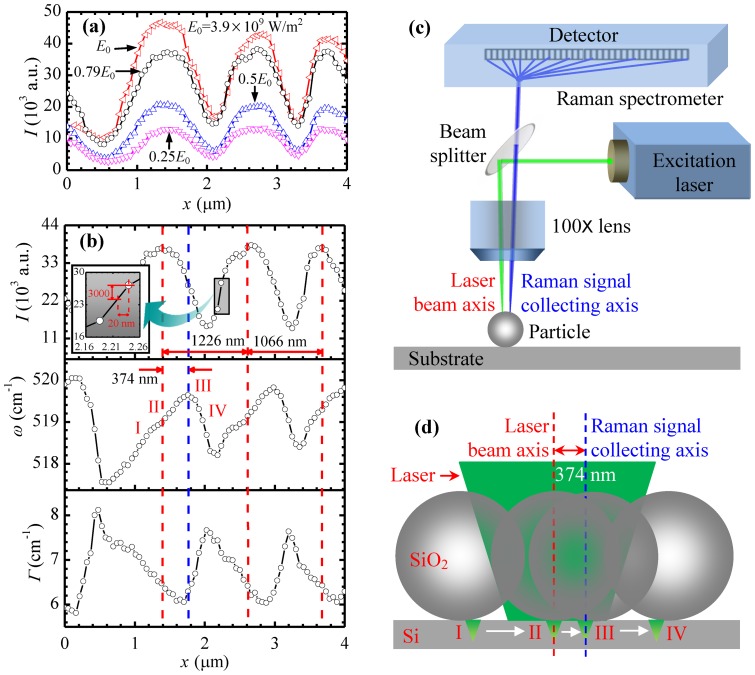
Nanoscale mapping for near-field heating under 1210 nm particles. (a) Raman intensity *I* variation for silicon along the mapping direction under particles of 1210 nm diameter with laser irradiation. (b) The *x* direction variation of Raman intensity *I*, Raman wavenumber *ω*, and linewidth *Γ* for silicon with laser irradiation of 3.1×10^9^ W/m^2^ (79%). (c) The distance between laser beam axis and Raman signal collecting axis of Raman spectrometer. (d) The position of a silica particle relative to the laser beam axis and Raman signal collecting axis to explain the observed Raman variation in space.

Four laser energy percentages of 25%, 50%, 79%, and 100% are used in our experiments, with the highest energy flux of 3.9×10^9^ W/m^2^. The integration time for acquiring the spectra is 2 s. Each Raman spectrum is measured 3 times automatically and averaged. Figure 3(a) shows the variation of silicon Raman intensity under 1210 nm particle induced laser focusing at four energy levels. The Raman intensity increases with the energy level. The highest Raman intensities (*I*_max_) are 1.30×10^4^, 2.08×10^4^, 3.83×10^4^ and 4.67×10^4^ for energy percentages of 25%, 50%, 79%, and 100%, respectively. As the variation trends of the structure are the same for different energy fluxes, here we only analyze the case for 3.1×10^9^ W/m^2^, of which the energy percentage is 79%. Other cases can be treated similarly. The Raman intensity *I*, wavenumber *ω* and linewidth *Γ* of silicon vary periodically along the *x* direction and are shown in Fig. 3(b). About three periods are observed within the travel range in the figure. The period length decreases from the left to the right. The difference may due to the diameter difference, the interspace among particles, and the backlash of the stage while moving. For the first 3/4 period, half of the period length is 906 nm, much longer than the average particle radius (560 nm). For the second and third periods, the period lengths are 1226 and 1066 nm, respectively, close to the average particle diameter (1120 nm). In the second period, the intensity difference between *I*_max_ and *I*_min_ is 2.37×10^4^, with a maximum intensity ratio (*I*_max_/*I*_min_) of 2.58. As shown in the inset of Fig. 3(b), within a distance of 20 nm in the sample moving direction, there is a Raman intensity difference of about 3000, and the intensity ratio within this distance is 1.12. This intensity difference can be distinguished by the Raman spectrometer in our experiment. As the intensity is the raw datum without any further processing, and it can reflect the morphology change in the surface of the sample, so it is the best quality to specify the imaging resolution. It is conclusive that the imaging resolution based on the Raman intensity difference can be down to 20 nm, although the step length in the experiments is 53 nm.

In the period, *ω* changes in a range from 518.2–519.6 cm^−1^, with a maximum shift of 1.4 cm^-1^. At position II in Fig. 3(b), *ω* is about 519.0 cm^−1^, where *I*_max_ is located. *ω* increases to the maximum at position III with a distance of 374 nm, then decreases to the minimum within 426 nm, and finally rises to 519.0 cm^−1^ again. *Γ* changes from 6.0 to 7.7 cm^−1^, with a maximum difference of 1.7 cm^−1^. The variation of *Γ* is contrary to that of *ω*. It first decreases from 6.4 to 6.0 cm^−1^, then increases to 7.7 cm^−1^, and finally decreases to 6.4 cm^−1^ again. The total increasing and total decreasing distances are not equal in a period for both *ω* and *Γ*, separately. There are three main reasons that can account for the difference. First, the beam axis of the excitation laser and the signal collecting axis of the Raman spectrometer are off a little (not exactly confocal). Figure 3(c) shows that the beam axis is not exactly parallel with Raman signal collecting axis. The varying angle of the scattered light coming into the spectrometer leads to a virtual spectral change in the detector. The transverse distance between the most excited Raman signal point and the Raman signal collecting axis on the particle is at sub-micron scale. When the sample moves relative to the laser, the variations of the *ω* and *Γ* curves are not symmetric. However, if the laser beam axis is coincident with the Raman signal collecting axis, the *ω* and *Γ* curves would be symmetric.

As the laser spot size is more or less the same with the particle diameter, it can cover about only one particle. Figure 3(d) illustrates four relative positions of a particle to the laser, corresponding to the four positions marked in Fig. 3(b). The red line shows the laser beam axis, and the blue line represents the signal collecting axis of the Raman spectrometer. When the particle is at position I, the laser irradiates the right part of the particle. The laser is focused on the substrate by the particle. As the laser center is of distance from the signal collecting center line, the Raman scattering signal, which comes back to the objective, is not at the laser focal plane. This out-of-focus effect leads to the variation of Raman signal. It would decrease the Raman wavenumber and broaden the linewidth as discussed before. At position II, the laser focal spot through the particle is coincident with the laser beam axis. Raman scattering signals are most excited. The Raman intensity reaches maximum. The laser focal point is near the Raman signal collecting axis but not coincident. As a result, the out-of-focus effect still exists. The Raman wavenumber keeps increasing and the linewidth continues decreasing when the particle moves towards position III. At position III, the laser focal spot is at the Raman spectrometer collecting axis. Raman scattering signals are accurately collected by the spectrometer. The Raman wavenumber and linewidth are at their extreme values because the collected Raman signals are from the focal plane of the Raman collecting optical path. There is no out-of-focus effect at position III. The Raman intensity is not quite high because part of the particle is outside of the laser beam. From position III to IV, the collected Raman signals become more out of focus. Thus, the Raman wavenumber goes down and linewidth broadens. In addition, the laser is focused only by part of the particle, so the Raman intensity is becoming weaker. Based on this analysis and the distance between the intensity peak and wavenumber peak [Fig. 3(b)] we conclude that the laser beam axis and the Raman signal collecting axis is off by about 374 nm under the 1210 nm particle near-field focusing. As the two axes are not exactly parallel, while the particle size varies, the distance between the two axes on the particle would be different. Under 400 nm silica particle focusing, the distance between these two focal centers becomes smaller, around 159 nm.

The second reason is that part of the Raman signals come from the silicon wafer under the spacing among particles. As the laser beam is pre-focused on the particles by the objective lens, the focal level for the silicon beneath the particles is at a higher position than the focal plane. This affects the Raman wavenumber and linewidth of silicon. The third reason may be due to the beam deflection caused by thermal expansion. The local silicon under the particle center is heated by the laser beam and expands. The heated silicon surface is no longer perpendicular to the propagation direction of the laser, which causes beam deflection. The beam deflection affects the Raman signal of silicon.

### De-Conjugation of Thermal, Stress, and Optical Effects

For the nanoscale imaging (Fig. 3) based on the Raman intensity, wavenumber, and linewidth, their variation against location reflects a combined effect of near-field optical heating, local stress, and optical field variation in space. Physically, it is possible to de-conjugate these three effects and obtain quantitative information about the nanoscale local stress and temperature. To do this, the Raman spectra for silicon under monolayer silica particles and for bare silicon are compared under four energy fluxes, respectively. The highest energy flux is 3.9×10^9^ W/m^2^, and the four energy percentages are 25%, 50%, 79%, and 100%. The integration time is 2 s. The Raman spectra at various sample positions and focal levels are obtained, and position II, as shown in Fig. 3, is selected to determine the thermal response of silicon. At this position, Raman intensity reaches its maximum value.

**Figure 4:**
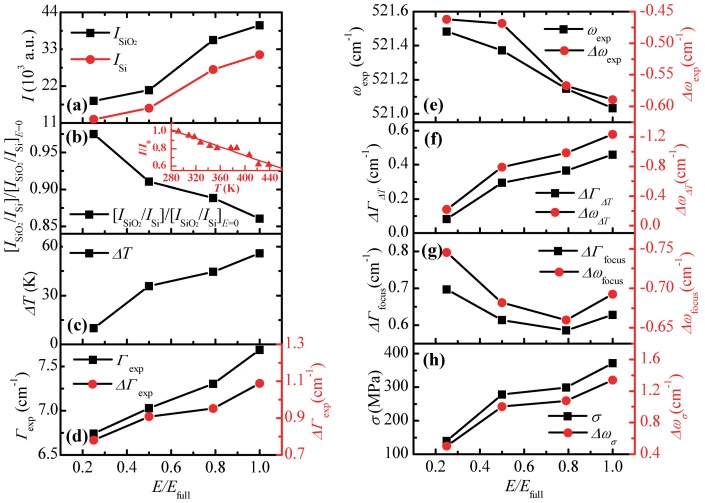
Temperature rise and thermal stress inside silicon under particle-focused laser irradiation. (a) How Raman intensity of silicon under 1210 nm silica particles (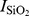) and that of pure silicon (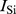) vary with energy percentage (*E/E*_full_). (b) Normalized Raman intensity ratio (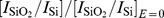) and (c) temperature rise (*ΔT*) versus energy percentage. The inset in figure (b) shows the linear relation between normalized Raman intensity of silicon (*I*/*I*_0_) and temperature with a slope of −0.00249 K^−1^. *I*_0_ is the intensity of silicon at 292.0 K. (d) Raman linewidth and (e) Raman wavenumber of silicon under particles and their differences with those of pure silicon. (f) Raman wavenumber and linewidth changes due to temperature rise. (g) Raman wavenumber and linewidth changes due to out-of-focus effect. (h) Thermal stress (*σ*) and Raman wavenumber change induced by stress under different laser energies.

First of all, by studying the Raman intensity variation against laser energy, the temperature rise under near-field heating is evaluated. The Raman intensity for silicon under silica particles (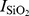) and that for bare silicon (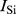) are shown in Fig. 4(a). 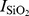 is higher than 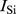 for each energy percentage because of the particle focusing. The intensity ratio 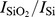 decreases linearly with the laser energy. Based on this ratio change against energy, extrapolation is conducted to determine the ratio at zero laser energy 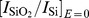. The normalized intensity ratio 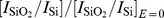 decreases with laser energy. From the decreasing trend, the temperature rise information can be extracted. The physics is as follows. There are three main factors that would affect the Raman intensity. Thus the Raman intensity can be expressed as 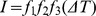 where *f*_1_ denotes the intensity change due to the system alignment; *f*_2_ represents the intensity change caused by the laser energy effect, which is proportional to the laser energy; and *f*_3_(*ΔT*) is the intensity variation induced by the temperature rise. For silicon with silica particles on its top, we have 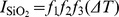. But for bare silicon, *_I_*si = *_f_*1*_f_*2, as the temperature rise is negligible here because of the large thermal conductivity of silicon. *_I_*si is acquired immediately after 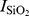, so *_f_*1 and *_f_*2 are the same for both 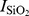 and *_I_*si separately. Thus, the intensity ratio is only relative to temperature rise: 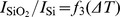. The Raman intensity of silicon reduces with the increase of temperature. This is because high temperature, which is caused by particle induced heating, changes the band structure in silicon, and it restricts the photon interactions necessary to generate Raman signals. The state density and energy of phonons increase as temperature rises, leading to a reduction of Raman intensity. In order to determine the thermal response inside silicon under laser irradiation, the temperature dependence of Raman intensity is needed. In our calibration, shown in the inset of Fig. 4(b), the normalized intensity *I*/*I*_0_ for bare silicon decreases with temperature, where *I*_0_ is the intensity of silicon at 292.0 K. The integration time is 2 s, and the laser energy is 8.6×10^8^ W/m^2^. The attained linear fitting slope for normalized Raman intensity against temperature is −0.00249 K^−1^ at temperatures from 290 to 440 K. Figure 4(b) shows that the normalized intensity ratio 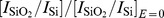 reduces when energy flux increases. Based on the relation between intensity and temperature, the temperature rise inside silicon due to particle focused laser heating is obtained. Figure 4(c) illustrates that *ΔT* increases from 10.0 to 56.1 K when the energy percentage goes up from 25% to 100%. The uncertainty of temperature rise can be evaluated according to uncertainty of intensity ratio at zero laser energy, which is about ±7.0 K.

In order to determine the thermal stress *σ* inside silicon, combined use of Raman wavenumber *ω* and linewidth *Γ* is necessary. When the temperature of the material increases, *ω* decreases and *Γ* broadens 29]. *Γ* has lower temperature sensitivity than *ω*. *Γ* is stress insensitive to the first order, while stress causes a shift in *ω* 15,30]. Considering the temperature rise and out-of-focus effect for silicon, the experimental linewidth difference can be expressed as *ΔΓ*_exp_  =  *ΔΓ_ΔT_* + *ΔΓ*_focus_, where *ΔΓ*_exp_ is the experimental linewidth broadening; *ΔΓ_ΔT_* is the linewidth change due to temperature rise; and *ΔΓ*_focus_ is the linewidth change because of the out-of-focus effect. For the experimental wavenumber softening, *Δω*_exp_  =  *Δω_ΔT_* + *Δω*_focus_ + *Δω_σ_*, where *Δω*_exp_ is the wavenumber difference between silicon under particles and bare silicon; *Δω_ΔT_* is the difference due to temperature rise; *Δω*_focus_ is the difference because of the out-of-focus effect; and *Δω_σ_* denotes the wavenumber shift induced by thermal stress. Figures 4(d) and (e) show the experimental Raman linewidth and wavenumber of silicon under particles and their differences from those of bare silicon. The standard deviations for linewidth and wavenumber in the data fitting are 0.06 and 0.02 cm^−1^, respectively. From our previous results, the linewidth of silicon increases linearly with temperature and the slope is 0.0082 cm^−1^/K, and the slope for the wavenumber against temperature is −0.022 cm^−1^/K 26]. Based on the temperature rise (*ΔT*) calculated from intensity [Fig. 4(c)], *ΔΓ_ΔT_* and *Δω_ΔT_* can be obtained according to the slopes [Fig. 4(f)]. So the linewidth difference due to focus effect is achieved by *ΔΓ*_focus_  =  *ΔΓ*_exp_ – *ΔΓ_ΔT_*. *Δω*_focus_ needs to be calculated from *ΔΓ*_focus_. The experiments to determine the relation between wavenumber and linewidth at different focal levels has been conducted in our lab 26]. The experimental data give a relation of *Δω*_focus_  =  −0.21055 −0.76742*ΔΓ*_focus_. Thus, the wavenumber difference due to focus effect is obtained and shown in Fig. 4(g). And the wavenumber shift induced by stress is given by *Δω_σ_*  = *Δω*_exp_ – *Δω_ΔT_* – *Δω*_focus_. A relation between the shift of Raman wavenumber *Δω_σ_* and the stress *σ* inside silicon has been developed with a proportionality constant of −3.6 cm^−1^/GPa 15]. This calibration is for bulk silicon. In our work, the heating region and stress existing region is at nanoscale (∼100 nm). Such size difference will not affect the validity of the calibrated relationship between stress and Raman peak shift. This is because in our work, the Raman signal collecting region is large enough for statistical average and to reflect the structure change (stress). So, the thermal stress *σ* inside silicon is acquired according to the relation. In Fig. 4(h), *σ* increases from 140 to 370 MPa as energy percentage of laser goes up from 25% to 100%. The uncertainty of thermal stress is about ±40 MPa, calculated from the uncertainty of *Δω_σ_*. Comparing Figs. 4(c) and (h), thermal stress goes up as local temperature rise increases, because the developing thermal stress is induced by the temperature gradient. The particle-focused laser beam heats up the silicon under the particle within a small area around 200 nm in radius. The localized heating of the beam causes thermal expansion in the heated area which is constrained by the nearby cold silicon. This constraint places a compressive stress on the heated region. The stress in silicon caused by the weight of particle is negligible. Assume the diameter of the contacting area between particle and substrate is 10 nm, then the pressure (*p*  =  *ρVg*/*A*) on the substrate is only about 260 Pa, where *ρ* is density of silica, *V* is the particle volume, *g* is acceleration of gravity, and *A* is the contacting area.

### Nanoscale Mapping for Near-Field Heating under 800, 400 and 200 nm Particles

**Figure 5:**
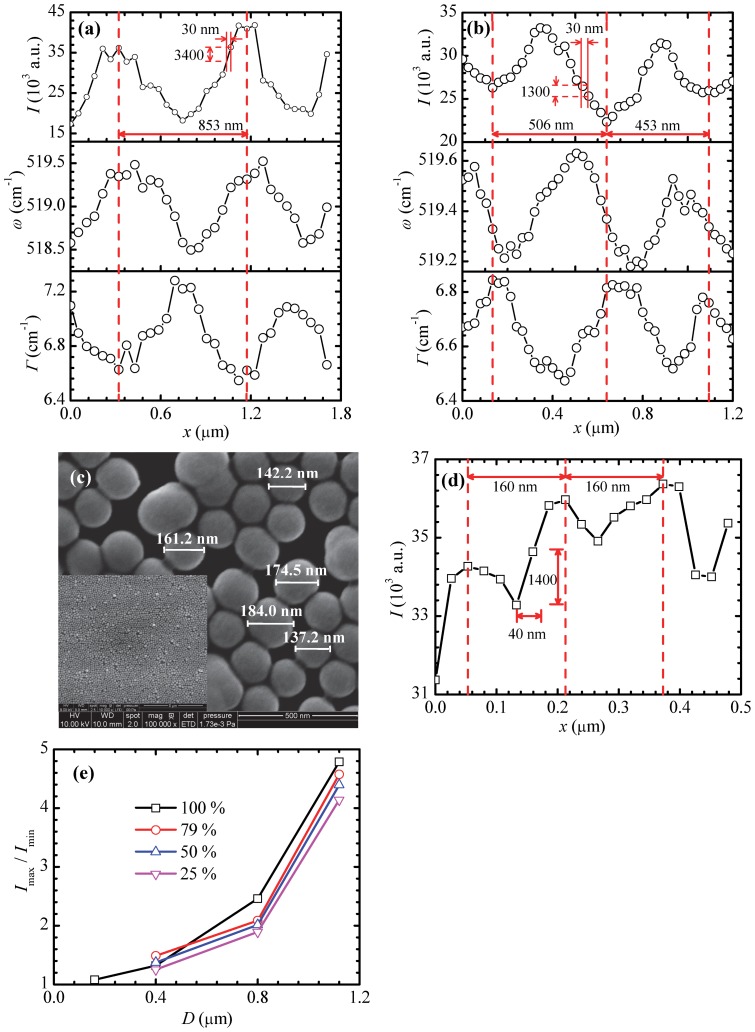
Nanoscale mapping for different sizes of particles. The Raman intensity *I*, Raman wavenumber *ω*, and linewidth *Γ* for silicon under particles of (a) 800 nm and (b) 400 nm diameters with laser irradiation. (c) SEM images of 200 nm particles on a substrate. The average diameter of the particles is about 160 nm. (d) The Raman intensity of silicon under particles of 200 nm diameter along the *x* direction. (e) The variation of maximum intensity ratio in silicon with particle size under four laser energy fluxes.

The experimental method for the 1210 nm case is employed to attain nanoscale imaging for the 800, 400 and 200 nm cases. The integration time is 2 s. Four energy levels are also used in the Raman mapping experiments for 800 and 400 nm cases. Similar mapping results are observed as in Fig. 3(a). Here we only discuss the cases under energy level of 3.1×10^9^ W/m^2^ (79%). The results for the 800 nm case are illustrated in Fig. 5(a). The total travel range along the *x* direction is 1.8 μm with a step of 53 nm. More than two periods are measured. The *I*_max_ is 2.24×10^4^, 2.82×10^4^, 4.18×10^4^ and 5.96×10^4^ for the four energy percentages of 25%, 50%, 79%, and 100%, respectively. For the 79% case with energy flux of 3.1×10^9^ W/m^2^, the second intensity period is 853 nm. The intensity difference and ratio of *I*_max_ and *I*_min_ are 2.27×10^4^ and 2.24, respectively. Within a distance of 30 nm in the *x* direction, the intensity difference and ratio are 3400 and 1.10, respectively, as shown in Fig. 5(a). It indicates that the resolution can be reached at about 30 nm in this case. In this period, *ω* varies from 518.5 to 519.5 cm^−1^, with a maximum shift of 1.0 cm^−1^. *Γ* varies in a range of 6.5–7.3 cm^−1^, with a maximum difference of 0.8 cm^−1^. The variation curves of *ω* and *Γ* along the *x* direction are not symmetric, and the reasons are similar with those for the 1210 nm case.

Figure 5(b) shows the variation of silicon Raman intensity along the *x* direction for the 400 nm case. The total travel range in the *x* direction is 1.2 μm with a step of 27 nm. The *I*_max_ is 1.40×10^4^, 1.95×10^4^, 3.36×10^4^ and 4.32×10^4^ for the four energy percentages of 25%, 50%, 79%, and 100%, respectively. In the case of 3.1×10^9^ W/m^2^, of which the energy percentage is 79%, the intensity period lengths are 506 and 453 nm. In the first period, the intensity difference between *I*_max_ and *I*_min_ is 1.09×10^4^, with a ratio of 1.49. Within a distance change of 30 nm in the *x* direction, the intensity difference and ratio are 1300 and 1.05, respectively, as shown in Fig. 5(b). In the period, *Γ* varies in a range from 6.5–6.9 cm^−1^. *ω* changes from 519.2 to 519.6 cm^−1^. The *Γ* and *ω* curves are not symmetric, either.

To explore the particle diameter limit of the far-field nanoscale imaging, 200 nm particles are used in the experiments. Figure 5(c) shows large areas of 200 nm monolayer particles on silicon. The average diameter of the particles shown in the SEM image is about 160 nm. Nanoscale imaging experiment is conducted along the *x* direction within a travel range of 500 nm with a step of 27 nm. The travel range covers about 3 particles. The laser energy flux is 3.9×10^9^ W/m^2^ (100%). The variation of the Raman intensity with *x* is shown in Fig. 5(d). The two distances between the highest intensities are both 160 nm, which agree well with the particle diameter observed under SEM. *I*_max_ is about 3.64×10^4^ with a *I*_max_/*I*_min_ of about 1.08 for the first period. The intensity change is about 1400 in a quarter of a period, which is of 40 nm distance, and the intensity ratio is about 1.04 within this distance. For this imaging, it is conclusive the imaging resolution can reach 40 nm. Figure 5(e) shows how the maximum Raman intensity ratio (*I*_max_/*I*_min_) varies with particle diameter under different energy fluxes. *I*_max_/*I*_min_ drops exponentially with the decrease of the particle size, and increases with the energy flux. As the particle size decreases from 1120 to 160 nm, *I*_max_/*I*_min_ reduces from about 4.8 to 1.1. With the decreasing trend, when the diameter of particles drops to 140 nm, it would be hard to tell the intensity difference within a period. The surface variation of a particle at diameter beyond 140 nm is able to differentiate using the confocal Raman spectrometer and microscope, although we cannot see it clearly under the microscope. The lateral resolution improves with the increase of the particle size. The best resolution is about 20 nm when the particle diameter is 1120 nm in our experiments. This technique can also be employed to detect nanoscale periodical surface variation of an object in the far-field.

### Physics behind Nanoscale Mapping

**Figure 6:**
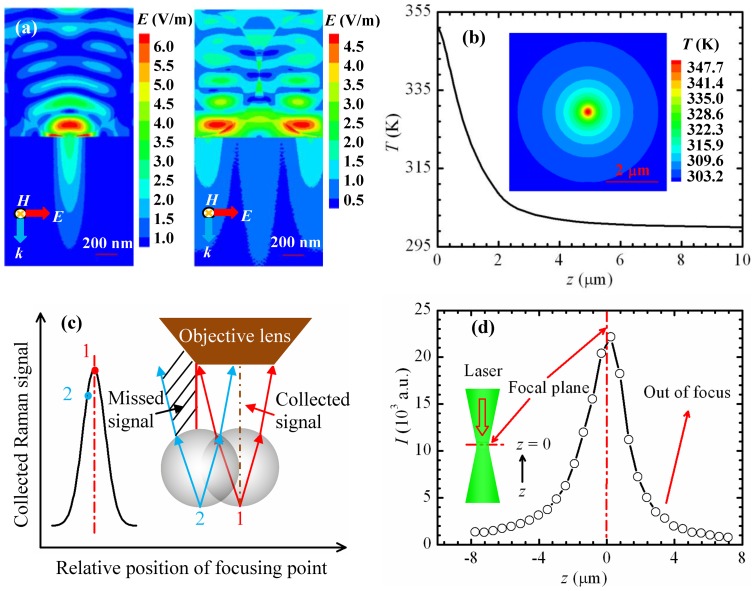
Modeling results and difference illustration. (a) HFSS modeling of a plane wave passing through a 1.21 μm silica sphere (*ε* = 2.13+ 0*i*) in air above a silicon substrate (*ε* = 17.22+0.428*i*). The amplitude of electric field is equal to the enhancement factor. In the left figure, the particle center is under the laser spot center. In the right figure, the particle center is at the fringe of the laser spot area. (b) Temperature profile inside a silicon substrate beneath a 1210-nm silica particle under laser irradiation. The inset shows the temperature distribution on the top of the substrate. (c) How the collected Raman signal varies with distance between the center of objective lens and laser focusing point in silicon. Position 1 represents the coincidence of the focusing point and the lens center, and position 2 shows a distance between them. (d) The variation of silicon Raman intensity with the laser focal level in the vertical direction.

When a laser beam irradiates a particle-substrate system, the laser is focused by the microparticle in a near-field region near the contacting point of the particle and the substrate. As the silica particle is transparent to green light, the particle absorbs little laser energy. Strong energy absorption occurs in the silicon substrate under the particle within a tiny elliptical zone near the surface. The optical field in silicon at the contacting point is the highest, and it attenuates from the surface to the inside of the substrate due to photon absorption. Such light absorption gives rise to a local temperature rise, and causes thermal expansion. The thermal expansion of heated silicon is constrained by the nearby cold silicon, which induces a compressive stress in the local region. To understand the mechanism of temperature and stress rise in the particle-substrate system, electromagnetic simulation is conducted with finite element analysis using the high frequency structure simulator (HFSS V14, ANSYS). Only the 1210 nm case is studied. Other cases can be treated similarly. In consideration of the amount of calculation and mesh density in HFSS, a quarter of the original model is employed. The remaining model is set to be symmetrical in both electric and magnetic directions. A plane wave (*λ*  = 532 nm) is incident normally from the top. In the experiment, the laser spot area is about the diameter size; only the particles in the spot area are under irradiation; no light irradiates the particles outside that area. To meet the experimental condition, only the parts of particles inside the laser spot area remain in the model. Other parts of particles outside the area are cut off to avoid receiving the plane wave. Perfect *H* and Perfect *E* symmetry boundaries are adopted at symmetrical planes. Absorbing (radiation) boundaries are applied for other boundary planes in the domain. The electric field amplitude of the incident wave is set to 1 V/m. Therefore, the near-field enhancement value is the same as the electric field amplitude of the scattered light. Two typical cases regarding to different laser-particle positions in an imaging period are simulated. Figure 6(a) shows the electric field distributions inside the substrate-particle system for the two cases. In the left case, the particle center is under the laser spot center, where the maximum enhancement is achieved in an imaging period. In the right case, the particle center is at the fringe of the laser spot, and the enhancement is the minimum in a period. The highest enhancement values for the two cases are 2.8 and 1.6 inside the substrate, and 6.4 and 4.8 inside the substrate-particle system. The maximum enhancement ratio of light intensity in the simulation is only 3.1, smaller than the maximum Raman intensity ratio in the experiments (4.8). The reasons for the difference will be discussed later. The laser focusing areas in silicon are right beneath the particles which are under laser irradiation, with a radius of about 200 nm.

The temperature distribution inside the silicon substrate is modeled using FLUENT (V12.0.1, ANSYS) based on the electric field distribution. The 1210 nm case with the highest energy and electric field enhancement is simulated in this work. As the temperature distribution inside silicon is symmetric, a quarter-cylinder computational domain with a radius of 5 μm and a height of 10 μm is considered in the simulation. Both vertical cross-sections use symmetric boundary conditions. Because the temperature field is measured at steady state, and the heat dissipation via radiation is small, so the top end surface of silicon is set as adiabatic. The peripheral and foot end surfaces of the domain and the initial temperature of the substrate are set at 300 K. The heat source is imported from the HFSS calculation results. The heat generation rate per unit volume can be calculated from 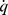  =  *Iβ*, where *β*  = 4*πκ*/*λ* is the absorption coefficient, *κ* is the extinction coefficient, *λ* is the wavelength of incident laser, and *I = _P_ = *0.5*cε*_0_ *nE* ^2^ is the laser intensity inside the silicon substrate, which is equal to the Poynting vector. The light speed in free space *c* = 3×10^8^ m/s, the vacuum permittivity *ε*_0_ = 8.854×10^−12^ F/m, the refractive index of silicon *n* = 4.15, and *E* (V/m) the time-average intensity of the electric field, which is acquired using HFSS. Other details of the temperature simulation can be found elsewhere [26]. Figure 6(b) shows the temperature profile inside silicon from the surface to the bottom under the particle. The inset illustrates the steady state symmetric temperature distribution on the surface of the silicon substrate. The calculated maximum temperature rise inside the silicon is 50.9 K, which is close to the experimental value (56.1 K).

For the 1210 nm particle case, the maximum enhancement ratio of light intensity in the simulation is only 3.1, smaller than the maximum Raman intensity ratio in the experiments (4.8). There are four main factors considered leading to the differences between experiments and simulation. The first factor is the relationship between the collected Raman signal and the distance between the objective lens and the focusing point, as shown in Fig. 6(c). The collected Raman signal is the strongest when the focusing point inside silicon is at the center of the objective lens; see position 1 in the figure. The signal decreases with the increase of the distance between focusing location and the lens center during the scanning process. Position 2 represents a situation that some Raman signals do not come into the lens through the particle. Instead, they are missed by the Raman spectrometer. So the amount of collected Raman signals reduces. As a result, the collected Raman intensity ratio between position 1 and 2 rises. Second, Raman intensity of silicon varies with focal level in the vertical direction, as shown in Fig. 6(d). The Raman intensity reaches maximum at the focal plane, and decreases with the distance between the sample and laser focal plane position in the *z* direction. So the maximum Raman intensity at the focal plane is much higher than that at an out-of-focus status. Third, the incident laser employed in the electric field simulation is a uniform plane wave, while in the experiments the laser is focused by a 100× objective lens before it irradiates the sample. Although the laser is still uniformly distributed in space, the direction of propagation has been changed. The laser should be focused in an even smaller region by the particles, which brings up a higher temperature rise in the focused region. Finally, during the simulation of electromagnetic field by HFSS, the dimensions of the computational domain are quite large. The grid is not fine enough, so the precision of the computational results may drop to a certain extent.

## Conclusion

In this work, far-field nanoscale imaging of near-field focusing, thermal and stress fields in a silicon substrate beneath silica particles was conducted for the first time using Raman spectroscopy. Imaging based on the Raman intensity decrease, Raman wavenumber shift, and linewidth broadening all reflected conjugated near-field focusing, thermal, and stress effects. Difference in the imaging based on these three parameters was largely induced by the non-coincidence between the laser beam axis and the signal collecting axis of the Raman spectrometer. Our detailed analysis revealed that such imaging can achieve a lateral resolution better than 20 nm (<*λ*/26). The nanoscale imaging capacity was fully demonstrated by studying the near-field focus under silica particles from 1210 nm down to 160 nm. Physical methodologies were developed to separate the near-field focusing, thermal, and stress effects and evaluate the temperature rise and local stress in particle-induced near-field focusing. Under 1210 nm silica particles, the temperature rise in the near-field focusing region in the silicon substrate reaches 56.1 K under a laser fluence of 3.9×10^9^ W/m^2^, and the local stress is 370 MPa. Our study under different energy levels revealed that the temperature rise and local stress increased almost linearly with the energy fluence. The electromagnetic and temperature fields inside the substrate-particle system were simulated to interpret the measured temperature rise with sound agreement. The imaging method can be used to detect nanoscale periodical surface variation of an object, and the thermal and stress variation under the surface in the far-field.
